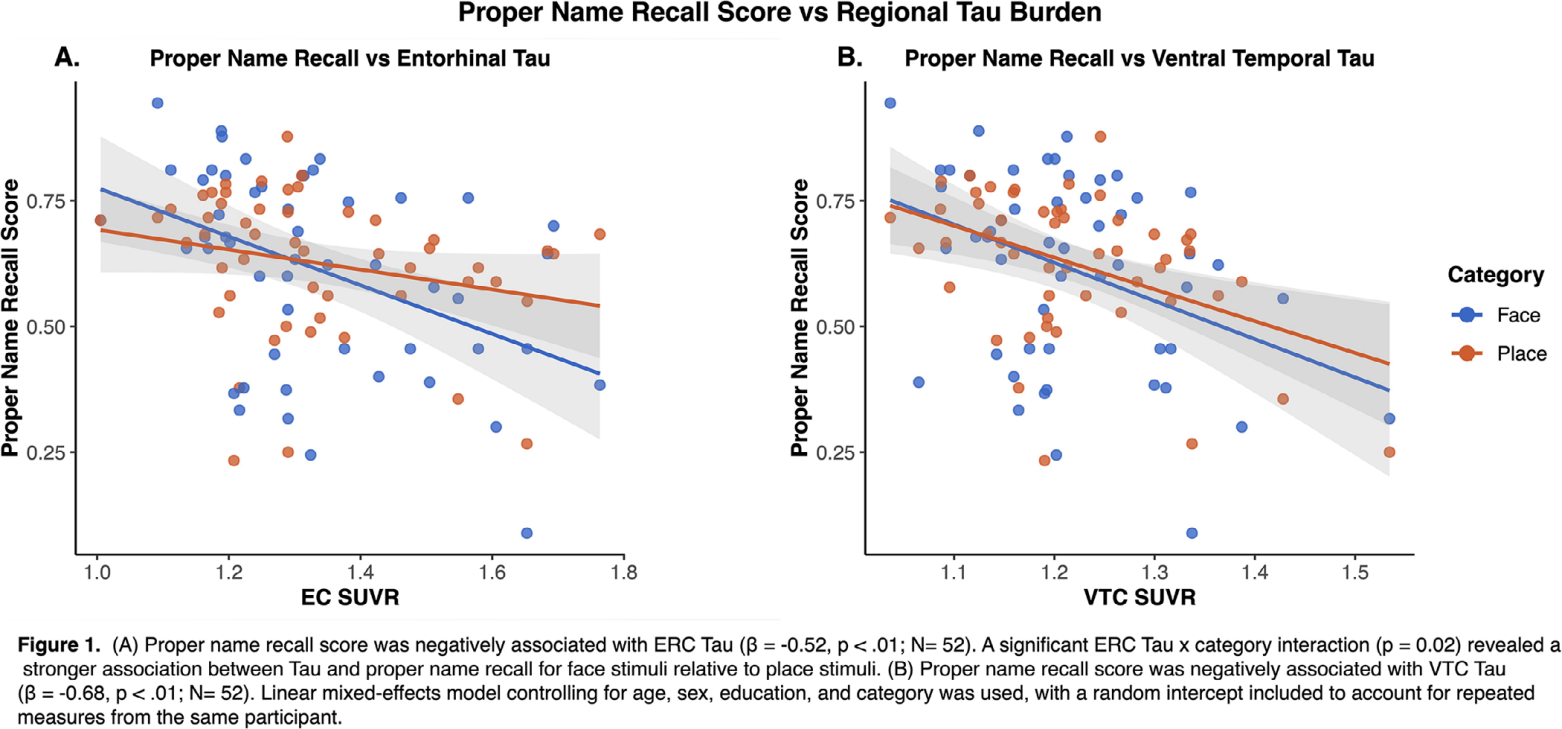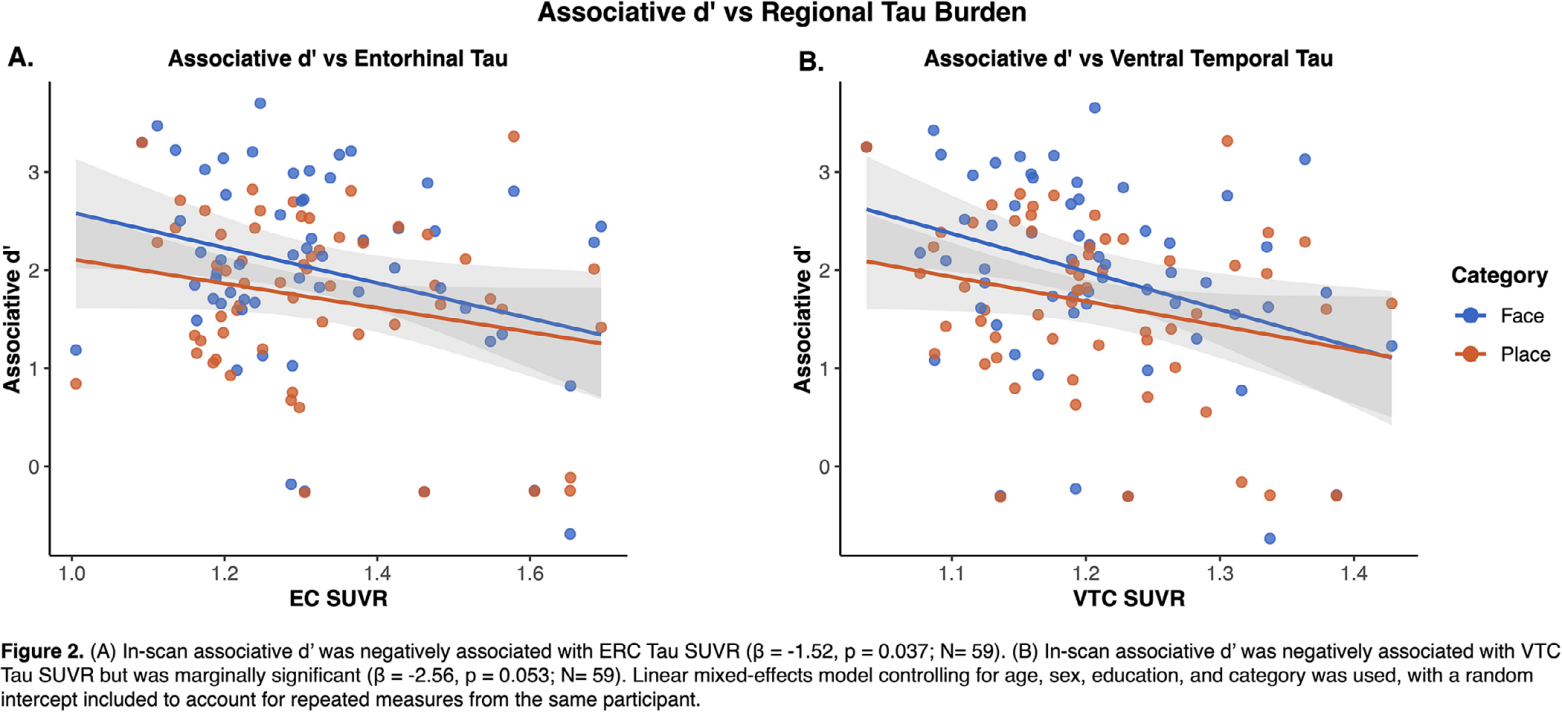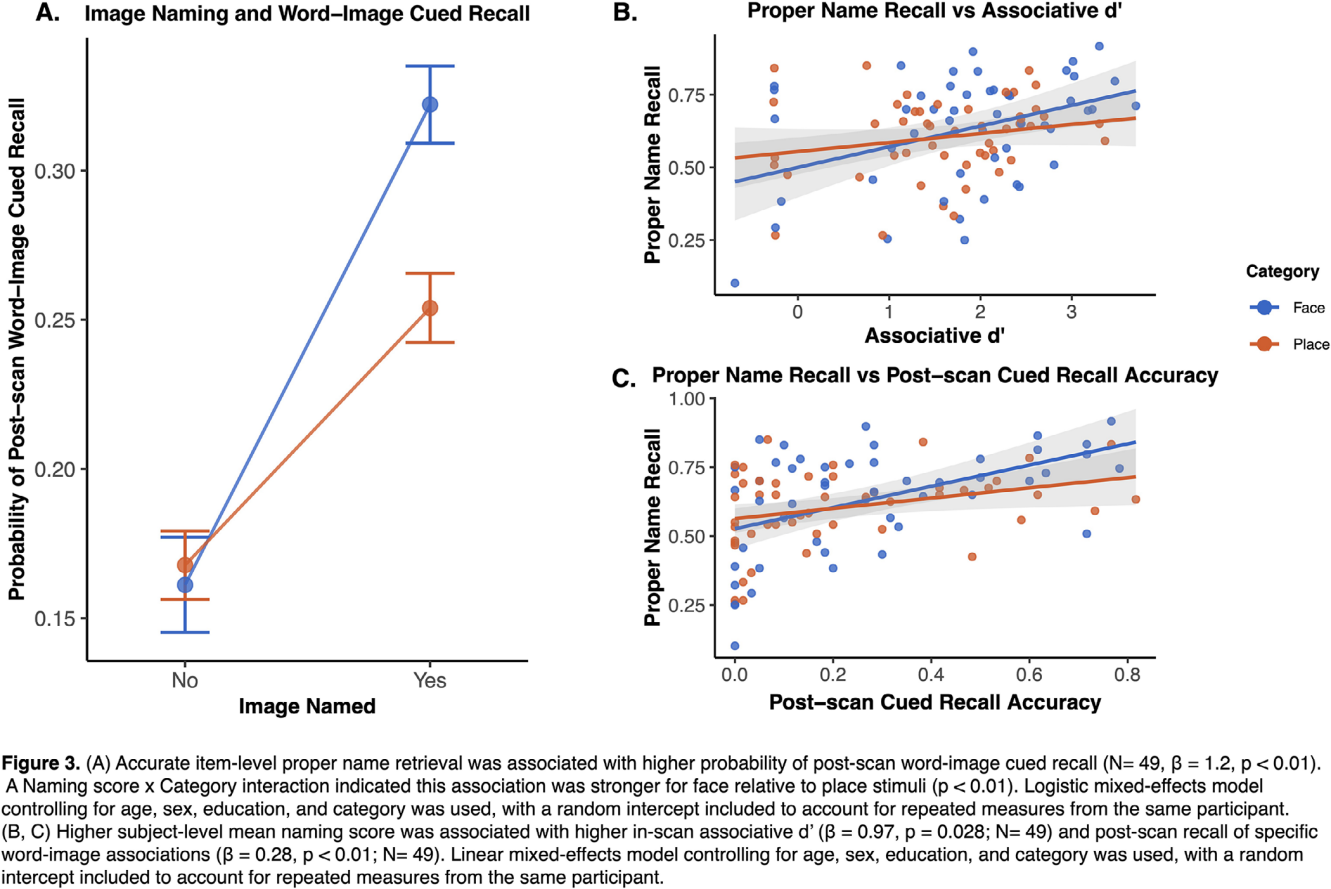# Associations between proper name retrieval, associative memory, and regional tau accumulation in a normal aging cohort

**DOI:** 10.1002/alz70862_109870

**Published:** 2025-12-23

**Authors:** Isha Sai, Alexandra N. Trelle, Jintao Sheng, Christina B. Young, Kyan Younes, Jennifer Park, Lucah Medina Guerra, America Romero, Hillary Vossler, Anthony D. Wagner, Elizabeth C. Mormino

**Affiliations:** ^1^ Stanford University, Stanford, CA USA; ^2^ Stanford University School of Medicine, Stanford, CA USA; ^3^ Wu Tsai Neuroscience Institute, Stanford, CA USA; ^4^ University of Oregon, Eugene, OR USA

## Abstract

**Background:**

Decline in episodic memory and proper name retrieval are common in aging and may be linked to early Alzheimer’s pathology in medial and ventral temporal cortex. We investigated associations between regional tau PET, associative memory, and proper name retrieval in a normal aging cohort.

**Methods:**

Participants were 59 cognitively unimpaired older adults (mean age = 75.97 ± 6.04 years, 61% female) from the Stanford Aging and Memory Study (SAMS). Participants completed a word‐picture (famous face or place) associative memory task concurrent with fMRI and a post‐scan cued‐recall test for word‐image associations. Proper name recall for face and place stimuli from the memory test was assessed on a separate visit 3.6 ± 3.82 months following the associative memory paradigm. Regional Tau accumulation was measured using 18F‐PI2620 PET, and standardized uptake value ratios (SUVRs) were extracted from the entorhinal cortex (ERC) and ventral temporal cortex (VTC; comprised of parahippocampal, inferior temporal, and fusiform gyrus). Linear and logistic mixed‐effects models assessed the associations between name recall, associative memory and tau PET controlling for age, sex, education, category, and random intercepts for subject.

**Results:**

Regional Tau in ERC (β = ‐0.52, *p* < .01) and VTC (β = ‐0.68, *p* < .01) was negatively associated with naming scores, with a stronger association for face stimuli (Tau x Category: *p* = 0.02; Figure 1). ERC Tau was negatively related to associative *d’* (in‐scan category memory) (β = ‐1.52, *p* = 0.037; Figure 2). Within individuals, item‐level analyses revealed a positive association between proper name recall and post‐scan test word‐image pair cued‐recall, with a stronger association observed for face stimuli (β = 1.2, *p* < .01; Figure 3A). Across individuals, naming score was positively associated with both associative *d’* (β = 0.97, *p* = 0.028; Figure 3B) and word‐image pair recall (β = 0.28, *p* < 0.01; Figure 3C).

**Conclusions:**

These findings suggest early tau burden is linked to impairments in episodic memory and proper name retrieval in aging. Furthermore, proper name retrieval is positively related to associative memory both within and across individuals, suggesting an influence of semantic knowledge on episodic memory.